# Keeping our balance in cerebellar ataxia: the contribution of neuroimaging to clinical investigation

**DOI:** 10.1590/0100-3984.2022.55.5e2

**Published:** 2022

**Authors:** Maria Clara Zanon Zotin

**Affiliations:** 1 Center for Imaging Sciences and Medical Physics, Department of Medical Imaging, Hematology, and Clinical Oncology, Faculdade de Medicina de Ribeirão Preto da Universidade de São Paulo (FMRP-USP), Ribeirão Preto, SP, Brazil. Email: czzotin@hcrp.usp.br; 2 Philip Kistler Stroke Research Center, Department of Neurology, Massachusetts General Hospital, Harvard Medical School, Boston, MA, USA

Ataxia is defined as the loss of coordinated voluntary muscle function and balance, usually manifesting as gait and speech impairment. These clinical symptoms are common to several neurological disorders with widely heterogeneous etiologies^(1-3)^. Cerebellar ataxia derives from lesions affecting the cerebellum or a combination of cerebellar and extracerebellar structures, such as the brainstem^(1,2)^. Patients with cerebellar ataxia may present with eye-movement disorders, speech abnormalities, together with abnormal limb movements, posture, and gait, as well as cognitive impairment, seizures, and autonomic deficits^(2,4)^.

Although there is a lack of robust epidemiological data, vertigo^(5,6)^ and movement disorders^(7-11)^ are among the most common neurologic complaints in emergency and outpatient settings and are a common reason for performing neuroimaging^(12)^. The overall prevalence of ataxia among children in Europe is estimated at approximately 26 cases per 100,000 population^(13)^. In a population-based study conducted in southeast Wales, the overall prevalence of late-onset cerebellar ataxia was estimated at 8.4 cases per 100,000 population for cases categorized as idiopathic and 1.8 cases per 100,000 population for those categorized as familial^(14)^. Worldwide, the reported prevalence of spinocerebellar ataxia ranges from 2 to 43 cases per 100,000 population^(15)^.

By analyzing the pattern of clinical features, we can predict the precise location of the lesions causing ataxia. For instance, according to the classical mediolateral division of the cerebellum, damage to midline cerebellar structures tends to cause gait and truncal ataxia, whereas hemispheric lesions usually cause homolateral appendicular ataxic symptoms^(1)^. Recent advances in the characterization of cerebellar networks, including their connections to the basal ganglia, thalamus, and cerebral cortex, have expanded our understanding of the functions of the cerebellum^(4)^. Therefore, a novel functional-anatomical approach to the cerebellar syndromes, based on the connectivity of the 10 cerebellar lobules (Larsell’s nomenclature), was developed; it categorizes the cerebellar syndromes as follows^(4)^: cerebellar motor syndrome, vestibulocerebellar syndrome, and cerebellar cognitive affective syndrome.

Although neurological examination continues to be successful in achieving anatomical localization, the underlying disorder causing ataxia cannot be diagnosed by clinical examination alone^(1)^. Symptoms can be widely variable and overlapping, a situation that is further complicated by extracerebellar neurological involvement. Limited clinical experience in the setting of rare ataxic disorders also reduces diagnostic precision^(1,14)^. Geographical location, ethnicity, and consanguinity directly influence the likelihood of specific hereditary disorders and add further complexity to the investigation of cerebellar ataxias^(16)^. With the ever-increasing number of genes implicated, the field of ataxiology has grown substantially in the last decades^(4)^. Therefore, the evaluation of a patient with ataxia currently involves multimodal strategies that strongly rely upon technological methods such as neuroimaging and genetic sequencing.

Various workflows have been proposed to guide the diagnostic workup of ataxic symptoms^(17)^. Neuroimaging evaluation through magnetic resonance imaging (MRI) is a common, essential step in all these workflows and plays two central roles in this investigation. First, conventional MRI should be used in order to exclude acquired causes of ataxia, such as stroke, neoplasia, infection, and malformation^(4,17)^. Once those have been ruled out, we are left with a wide range of neurodegenerative hereditary or sporadic ataxias, with neuroimaging findings that are fairly nonspecific and often overlapping. In this context, the precise causative disorder can rarely be defined by clinical and neuroimaging findings alone. Therefore, the second role of MRI is to narrow the list of potential diagnoses and guide more appropriate genetic testing^(17)^. In addition, advanced MRI techniques, such as diffusion tensor imaging/tractography, structural volumetric assessment, and functional MRI, may contribute to assessing the integrity of cerebellar connectivity in ataxic syndromes and could provide potential outcome measures for future clinical trials and for the follow-up of patients with ataxic symptoms^(4,18)^.

The pictorial essays authored by Jarry et al.^(19,20)^, published in the previous issue of Radiologia Brasileira, provide a valuable illustrated guide of common and uncommon neuroimaging manifestations of ataxia for radiology trainees. The authors reviewed the archives of a tertiary care hospital in Brazil and depicted acquired conditions and neurodegenerative causes of ataxia observed in this population. It is noteworthy that the authors did not intend to exhaust the subject of neuroimaging findings in cerebellar ataxia. They did, however, succeed in providing an overview of the disorders radiologists and neurologists are likely to encounter, especially among patients in Brazil. For instance, the authors provide several examples of acquired conditions that must be ruled out through neuroimaging during the investigation of ataxia. Such conditions include infections (tuberculosis, cryptococcosis, and an atypical presentation of progressive multifocal leukoencephalopathy), vascular complications (stroke and venous thrombosis), inflammatory diseases (neuro-Behçet’s disease), intoxication (with phenytoin), neoplasms (Lhermitte-Duclos disease, medulloblastoma, pilocytic astrocytoma, and ependymoma), and malformations (Dandy-Walker malformation). Finally, the authors depict the characteristic imaging features of three degenerative disorders^(19,20)^: progressive ataxia and palatal tremor; Friedreich’s ataxia; and Machado-Joseph disease. By exemplifying some of the most relevant neuroimaging findings related to ataxic symptoms, these essays could help radiologists exclude acquired disorders and narrow the differential diagnosis of hereditary cerebellar ataxia.
